# Epizootology and Molecular Diagnosis of Lumpy Skin Disease among Livestock in Azerbaijan

**DOI:** 10.3389/fmicb.2016.01022

**Published:** 2016-06-29

**Authors:** Shalala Zeynalova, Kliment Asadov, Fizuli Guliyev, Mahira Vatani, Vidadi Aliyev

**Affiliations:** ^1^Virology Department, Republican Veterinary LaboratoryBaku, Azerbaijan; ^2^Sector for Epidemiological Control, Treatment, and Prevention of Especially Dangerous Pathogens, State Veterinary Control ServiceBaku, Azerbaijan; ^3^Jalilabad Rayonal Veterinary OfficeJalilabad, Azerbaijan

**Keywords:** lumpy skin disease, pox virus, cattle, Azerbaijan, PCR

## Abstract

Lumpy skin disease (LSD) is a viral disease of livestock that can cause cutaneous and internal lesions, affecting milk production, hide quality and in some cases death of the infected animal. After an outbreak in neighboring Iran, a working group from the Azerbaijan State Veterinary Control Service was sent to the border rayons (administrative districts) to determine if any cattle in southern Azerbaijan were infected. The Rayonal Veterinary Offices were contacted to look for and report any cases of LSD in their rayons. Animals exhibiting clinical signs consistent with LSD infection were first observed in the rayon of Bilasuvar and more cases were subsequently identified in Jalilabad, Ujar, and Aghdash rayons. Samples were collected from blood, and/or lesions of suspected infected animals and internal organs of cattle that died and were tested at the Republican Veterinary Laboratory in Baku using real-time polymerase chain reaction (PCR). From June to November 2014, 2,762 cattle in Azerbaijan were reported to have clinical signs or gross necropsy lesions consistent with LSD. Of 269 samples tested for LSD virus by real-time PCR, 199 (74%) were positive. A total of 33 cattle died, which was 1.2% of those exhibiting clinical signs of disease. Samples from nodular cutaneous lesions were more frequently positive by PCR and had higher concentrations of virus than blood and pooled internal organ samples. Preventative measures including movement restrictions, vector control and vaccination were put into place to slow the spread of disease. Ongoing surveillance should continue as environmental persistence of the virus may lead to further outbreaks of disease.

## Introduction

Lumpy skin disease (LSD) is a disease of livestock caused by lumpy skin disease virus (LSDV), a DNA virus belonging to the genus *Capripox* in the *Poxviridae* family. Although other strains of *Capripox* infect sheep and goats, LSDV is associated with cattle (Davies, 1981). Lumpy skin disease was first recorded in Zambia in 1929, and then spread throughout southern Africa and north to Sudan. It was first diagnosed outside of Africa in Israel in 1989 and in subsequent years, cases were reported in Bahrain, Kuwait, Oman, Yemen, Lebanon, and Jordan (Wainwright et al., 2013). The disease is characterized by fever and nodular lesions on the skin, mucous membranes, and internal organs (Prozesky and Barnard, 1982; OIE Terrestrial Manual, 2010). Reduction in milk production, damaged hides, temporary or permanent sterility, and/or death of infected animals may occur, resulting in significant economic consequences in affected countries (Sajid et al., 2012; Alemayehu et al., 2013). Severity of clinical signs depends on the strain of the virus and breed of infected cattle (OIE Terrestrial Manual, 2010). Transmission is primarily by indirect contact via biting insects (Kitching and Mellor, 1986; Chihota et al., 2001; Magori-Cohen et al., 2012). The average incubation period of LSDV is six to nine days (OIE Terrestrial Manual, 2010). The average mortality rate of the disease is approximately 10%, although the mortality rate is often higher in secondary infections (OIE Terrestrial Manual, 2010).

Azerbaijan is situated on the Caspian Sea and shares a border with Russia to the north, Georgia to the northwest, Armenia to the west, and Iran to the south. Azerbaijan is divided into rayons (or administrative districts), and cities. Each rayon and city has a Rayonal Veterinary Office (RVO) that provides veterinary and epidemiologic support for the control of animal diseases in the rayon. Each rayon also has field veterinarians stationed throughout the rayon who report to the RVO. Each RVO reports any cases of reportable diseases to the State Veterinary Control Service (SVCS).

Outbreaks of LSD were reported to the OIE in Turkey and Iraq in late 2013 and in Iran in early 2014 (OIE World Animal Health Information Database [WAHID]; Wainwright et al., 2013; European Food Safety Authority [EFSA], 2015). The objective of the investigation was to determine if LSDV had spread into Azerbaijan from neighboring countries reporting cases. In May 2014, an outbreak response team was sent from the SVCS in Baku to Azerbaijan’s southern border area to examine cattle and to make control and prevention recommendations. This paper describes the first confirmed cases of LSD in Azerbaijan in 2014 (OIE, 2014) as well as the diagnostic methods used and control and prevention measures applied.

## Materials and Methods

### Affected Areas and Populations

**FIGURE 1:**
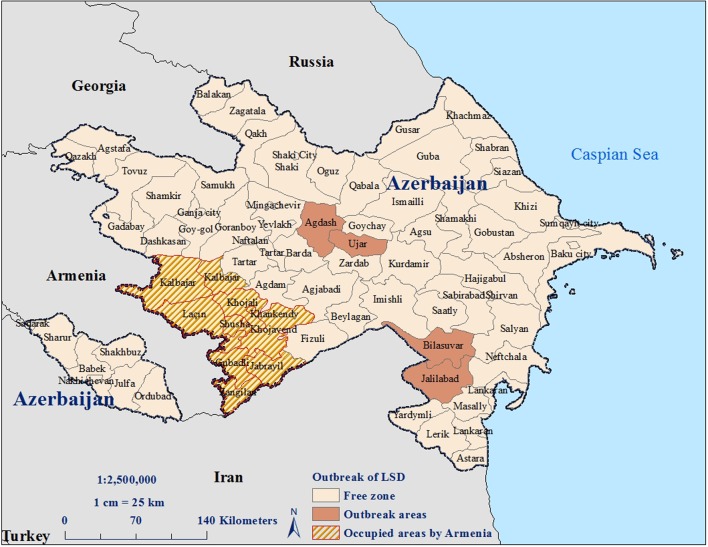
**Rayons with suspect or confirmed LSDV infections in cattle reported during 2014**.

In May 2014, a working group composed of clinicians and veterinary epidemiologists from the SVCS and local field veterinarians investigated small cattle farms in Bilasuvar rayon after reports of LSD were reported in the neighboring country of Iran. Bilasuvar is a common area of animal movement near the Iranian border. The veterinary epidemiologist from the RVO in Bilasuvar asked 35 field veterinarians stationed throughout the rayon to visit all farms in their areas to inquire about any suspected cases of LSD. A registration system of farms was not in place at the time, so identification of farms was based on the local knowledge of the field veterinarians. Cattle exhibiting clinical signs consistent with LSD were identified in the village of Amankend. Upon confirmation of suspect cases, every RVO in Azerbaijan was notified of the outbreak. In turn, the RVOs instructed field veterinarians across the country to report any suspect cases and to collect samples to be sent to the Republican Veterinary Laboratory for confirmatory testing. If positive cases were observed, veterinarians were instructed to visit those farms daily until the cases resolved or the animals died. Total case numbers were shared with the SVCS on a monthly basis. Cases of presumptive LSD were subsequently reported in the region of Jalilabad, which also borders Iran, and in Ujar and Aghdash, which are more centrally located within the country but lie along major roadways that are connected to southern Azerbaijan (**Figure 1**).

### Animal and Tissue Sampling

Field veterinarians were responsible for collecting biological specimens from affected animals according to established SVCS protocols and guidance provided by the SVCS at the time of the outbreak. Samples were sent to the Virology Department at the Republican Veterinary Laboratory for testing. A skin scrape of nodular skin lesions and 5 mL blood samples in EDTA were collected from the necks and heads of affected animals. Necropsies were performed by field or RVO veterinarians on 33 dead cattle exhibiting lesions consistent with LSD; small portions of lung, kidney, liver, and heart were collected from these animals for testing.

Samples were prepared according to SVCS protocol #475 (State Veterinary Control Service of Azerbaijan, 2008). Skin samples were placed into phosphate buffered saline (1X PBS) for one hour before DNA extraction. A piece (approximately 1.3 cm^2^) of each of the internal organs (liver, kidney, heart, and lung) was cut into small pieces using scissors and pooled together (up to 5 g total). Samples (skin or pooled internal organs) were homogenized using a mortar and pestle with a small amount of ground glass and 8 mL 1× PBS. Once homogenized, the slurry was decanted into 15 mL conical tubes and centrifuged at 8,000 rpm for ten minutes. DNA was extracted from 140 μL aliquots of the resulting supernatant using DNeasy Blood & Tissue Mini Kits (Qiagen, USA) according to the manufacturer’s instructions. These kits were also used to extract DNA from blood samples. Mortar and pestles were disinfected with bleach between samples. The study described was an example of outbreak surveillance conducted with approval of the Azerbaijan State Veterinary Control Service (SVCS) Scientific Committee, which reviewed the activities for scientific and ethical concerns for the use of animals.

Appropriate biosafety protocols were followed during sampling and laboratory work to ensure personnel, environmental and animal safety. Personnel wore personal protective equipment (PPE) and all instruments were disinfected between samples. Waste materials were properly treated and discarded.

### Real-Time PCR Testing

A real-time PCR assay was used to rapidly diagnose cases. Specific forward and reverse capripox primers were used as described by Balinsky et al. (2008); sequences are listed in **Table 1.** Primers, probe and positive template control materials were provided by Dr. Ketan Patel of the Naval Medical Research Center in Ft. Detrick, Maryland.

**Table 1:** Primer, probe, and positive template control sequences for the LSDV real-time PCR assay.

Primer/probe/PTC	Sequence
Forward primer	5′-TCC-GAG-CTC-TTT-CCT-TAC-TAT-3′
Reverse primer	5′-TAT-GGT-ACC-TAA-ATT-ATA-TAC-GTA-AAT-AAC-3′
Probe^a^	5′6FAM-CAATGGGTAAAAGATTTCTA - MGBNFQ 3′
Positive Template Control	5′ ATG GCG ATG TCC ATT CCC TGA CCA ATG GGT AAA AGA TTT CTA TCG TAA CAG ATG AAA GAG CAA GCT ACT ATT CCT CAC GGA AAT GAA ATG CTT C 3′


*^a^Fluorescent dye abbreviation: 6FAM = 6-Carboxyfluorescein; MGBNFQ = Minor groove binding, non-fluorescent quencher.*

PCR master mix was prepared using Taq polymerase, 10× PCR buffer, 50 mM MgCl_2_, and 10 mM dNTPs according to the protocol described by Balinsky et al. (2008). A total of 5 μL of extracted sample DNA or template controls were added to 15 μL of the prepared master mix for a total volume of 20 μL. The reaction was run on a LightCycler 2.0 PCR instrument (Roche Diagnostics, Germany) using the thermocycling conditions described by Balinsky et al. (2008). Before sample testing, the positive template control (PTC) was serially diluted from 1 pg/μL to 0.001 fg/μL and run on the R.A.P.I.D. PCR instrument (BioFire Defense, Salt Lake City, UT, USA). The resultant cycle threshold (*C*_T_) vs. log of the PTC concentration graph gave a slope of 3.76, corresponding to 92% amplification efficiency (with 100% doubling every cycle); this was sufficient for continued testing of samples. Based on the standard curve, PTC at a concentration of 1 fg/μL was used in subsequent measurements.

In order to determine the best sample to collect for testing of future cases, a comparison of PCR results was undertaken in the 33 animals that died where three sample types were tested including nodular lesions, blood and pooled internal organ samples as well as in the 27 animals that had paired blood and nodular lesion samples.

### Control Measures

Treatment of sick animals varied by case, but typically included disinfection of cutaneous lesions using iodine and treatment of secondary bacterial infections with sulfanilamide. All field veterinarians wore PPE while on animal premises and while handling animals including disposable gowns, rubber boots, gloves and head covers. All reusable instruments were disinfected between premises and waste materials were properly treated and discarded. Preventative measures were enacted, including movement control of animals as well as restriction of vehicle access to affected farms. Farms with affected animals were visited daily by the field veterinarians until the cases either died or recovered. Neighboring countries were notified of the presence of LSD in Azerbaijan and vaccines were ordered for a targeted vaccination campaign.

## Results

### Affected Area and Populations

**FIGURE 2:**
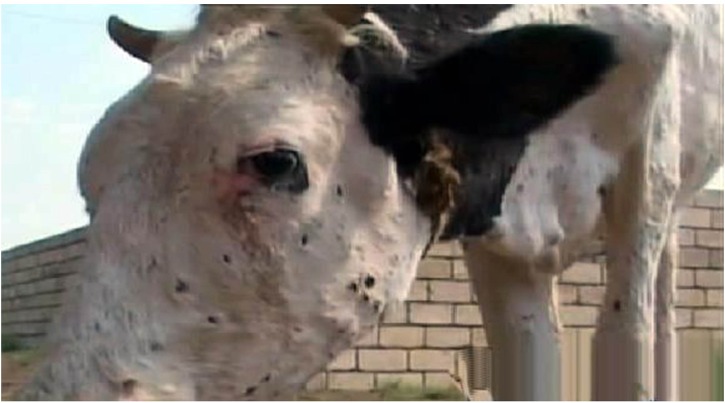
**Nodules on head area**.

**FIGURE 3:**
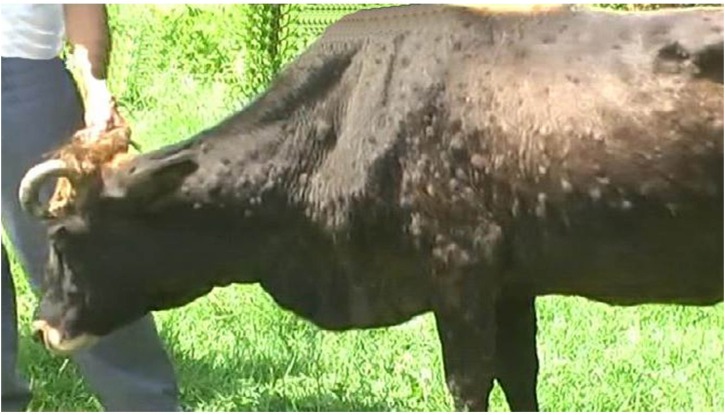
**Nodules on neck and abdominal area**.

Cattle exhibiting clinical signs consistent with LSD were reported in Bilasuvar, Jalilabad, Ujar, and Aghdash rayons (**Figure 1**). Affected cattle refused food and exhibited fevers, purulent oculonasal discharge, and malaise. In some cases, red, firm nodules were observed along the neck and abdomen of the animal, with degenerative changes noted on the skin surface around the nodules, such as necrotic areas, edema, and exudate (**Figures 2** and **3**). A pulmonary form resulting in shortness of breath was also documented, with the majority of cases reported in October 2014. Death in the pulmonary cases was presumed to have resulted from asphyxia. Lung congestion and nodules throughout internal organs were often observed during necropsies.

A total of 2,762 cases were reported and 33 cattle (1.2%) died (**Table 2**). Overall, about 6.5% of cattle in the affected villages were reported as positive to the SVCS with a 95% confidence interval of 6.2–6.7% (**Table 2**). A total of 14% of farms in those villages reported at least one case of LSD. Cases were reported in June, July, October, and November 2014 (**Table 2**).

**Table 2:** Clinical diagnosis of LSD in cattle by village and farm.

Rayon	Date of outbreak onset	Date of last case in village	Village	Estimated # of cattle	# Suspected cases	Apparent morbidity % (95% CI)	# Deaths	Apparent case fatality (%)	# Farms in village	# of Farms with LSD	% of Farms affected (95% CI)
Bilasuvar	June 07, 2014	June 21, 2014	Amankend	926	128	13.8 (11.7-16.2)	0	0	39	4	10.0 (3.3-25.1)

Jalilabad	July 08, 2014	July 24, 2014	Alar	3,142	114	3.6 (3.0-4.3)	0	0	32	4	12.5 (4.08-30)

Aghdash	October 04, 2014	October 25, 2014	Pirkeke	3,863	126	3.3 (2.7-3.8)	2	1.6	43	8	18.6 (9-34)
	October 04, 2014	November 01, 2014	Kukel	967	123	12.7 (10.7-15.0)	2	1.6	44	7	15.9 (7.15-30.6)
	October 04, 2014	October 22, 2014	Arabkendi	1,299	127	9.8 (8.2-11.5)	2	1.6	35	6	17.1 (7.16-34.3)
	October 15, 2014	November 05, 2014	Shakili	901	124	13.7 (11.6-16.2)	1	0.8	31	5	16.1 (6.1-34.4)
	October 05, 2014	November 29, 2014	Shansabd	1,342	137	10.2 (8.7-11.9)	3	2.2	44	7	15.9 (7.15-30.6)

Ujar	October 02, 2014	October 25, 2014	Elekend	3,519	287	8.1 (7.3-9.1)	3	1.0	31	5	16.1 (6.1-34.47)
	October 02, 2014	October 28, 2014	Leke	4,884	244	5.0 (4.4-5.6)	4	1.6	48	6	12.5 (5.1-26)
	October 03, 2014	November 01, 2014	Yuxarishilyan	4,921	276	5.6 (5.0-6.2)	3	1.1	51	8	15.7 (7.5-29.1)
	October 03, 2014	October 30, 2014	Alpoud	4,670	271	5.8 (5.2-6.5)	5	1.8	48	5	10.4 (4.53-22.1)
	October 03, 2014	October 25, 2014	Tezeshilyan	4,772	283	6.0 (5.3-6.6)	2	0.7	54	6	11.1 (4.6-23.3)
	October 04, 2014	November 02, 2014	Qaraqumlar	3,878	268	6.9 (6.1-7.7)	3	1.1	49	8	16.3 (7.8-30.2)
	October 05, 2014	October 27, 2014	Boyad	3,674	254	6.9 (6.1-7.8)	3	1.2	43	6	14.0 (5.8-28.6)

	**TOTAL**			**42,758**	**2,762**	**6.5 (6.2–6.7)**	**33**	**1.2**	**592**	**85**	**14.3 (11.7–17.5)**


### Real-Time PCR Testing

A total of 269 samples were tested by real-time PCR for the presence of LSDV from 176 animals, including 130 skin samples, 106 blood samples, and 33 internal organ pools (**Table 3**). A total of 199 (74%) samples were positive by PCR. All skin lesions tested were positive and had lower *C*_T_ values than blood or organ samples, suggesting higher concentrations of virus. Blood had the highest average *C*_T_ value and was least likely to be positive, suggesting lower concentrations of virus.

**Table 3:** Real-time PCR testing results.

Type of Sample	# Tested	# Positive	% Positive	Average *C*_T_ value	Standard deviation of *C*_T_ values
Skin Lesion	130	130	100	19.3	1.10
Blood	106	42	40	29.4	1.39
Internal Organ pool	33	27	82	22.9	0.65

**TOTAL**	**269**	**199**	**74**	**–**	**–**


*Samples are from 176 animals (33 were tested for all three samples, 27 had skin lesion and blood samples submitted while the remaining only had skin or blood tested).*

In the 33 animals that died, nodular lesions tested positive in all 33 cases (100%); 27 of 33 (82%) pooled internal organ samples tested positive while only 13 of 33 (39%) blood samples tested positive. Paired blood and nodular lesion samples were submitted for 27 suspect cases. Of these, all 27 nodular lesion samples (100%) were positive while only 11 blood samples (41%) tested positive.

### Control Measures

Overall, the majority of suspected infected cattle recovered, although it is unclear which, if any, treatment regimens contributed to recovery. All affected farms were instructed to restrict animal movement off the farm for 30 days from the time the last case was identified. Ectoparasiticides were applied to healthy ruminants on the infected farms and on surrounding farms where outbreaks occurred. One of three locally available ectoparasiticides was used to spray animals, including Ektosan (Brovafarma Ltd, Ukraine), Blotic 7% Emulsion (Topkim, Turkey) or Butox (MSD Animal Health, India). Dilutions were made according to manufacturer’s recommendations and farmers were asked to apply the ectoparasiticide twice weekly. After the outbreak, two million doses of live sheep and goat pox vaccine (Poxvac, Vetal Company, Turkey) were purchased. In 2015, a targeted 5-year vaccination campaign was initiated to control the spread of this disease in Azerbaijan. A total of 1.6 million cattle in the affected rayons, neighboring rayons, and rayons on the southern Azerbaijan border were vaccinated in 2015 with some vaccine held in reserve in the event of additional outbreaks. Cattle 3 months of age and over were included in the campaign with a focus on animals that migrate to summer pastures. For 2016–2019, approximately 15 million cattle are planned to be vaccinated throughout the country annually with 9 million cattle in high risk areas being vaccinated twice a year.

## Discussion

Although sheep and goat pox is considered endemic in Azerbaijan and the SVCS routinely conducts vaccinations against these diseases, LSDV had not been identified in Azerbaijan before the 2014 outbreak. Biting insects are thought to be responsible for transmitting this disease (Salib and Osman, 2011; Magori-Cohen et al., 2012) and may have introduced LSDV to the Bilasuvar region after crossing the border or being transported by vehicles into the rayon. Notably, the onset of this outbreak in early summer overlapped with periods of peak biting insect activity. It is also possible that the virus was introduced through the migration or movement of animals into Bilasuvar from an infected area, and was subsequently transmitted by direct or indirect contact. In support of this theory, Tuppurainen and Oura (2014) suggest that the infected animals could have originated in one of several nearby countries with LSD, such as Iran, Iraq, or Turkey. There is no conclusive evidence of either method of transmission. None of the farmers from where the first cases were observed reported animal movement from Iran or other LSD positive countries. The spread of infection within Azerbaijan may have resulted from movement of sale animals from Bilasuvar to the more northern rayons of Ujar and Aghdash. Farmers in the northern areas reported purchasing animals from the southern Azerbaijan rayons of Bilasuvar and Jalilabad, although specific farms were not identified. Additional characterization of the virus by sequencing may help to better determine the source of this outbreak. LSD outbreaks have since been reported to the OIE in Greece, Armenia, Georgia and Russia.

Overall, 6.5% of susceptible cattle in the affected areas were reported as having LSD and 1.2% of suspected infected cattle died during the 2014 outbreak in Azerbaijan. Official animal case definitions were not in place in Azerbaijan at the time of the outbreak, but have since been developed and formally adopted for future reporting purposes. It should be noted that not all suspected cases of LSD were confirmed to be infected by PCR, nor were other factors considered that may have caused animals to be more or less likely to die from infection. The apparent morbidity and case fatality rates reported from this outbreak are also subject to reporting biases as the estimates relied on passive surveillance. Variable mortality rates and case fatality rates for LSD have been observed in other countries. Mortality rates in an outbreak in Oman reached 13.6 and 15.4% in two locations (Tageldin et al., 2014), while a mortality rate of 2% was reported among cattle in six feedlot operations in Ethiopia (Alemayehu et al., 2013). An analysis of active and historic outbreaks of LSD in Ethiopia revealed mortality rates between 3.4 and 5.9% (Ayelet et al., 2014). In Jordan, a study assessing the efficacy of vaccination reported 10% mortality among unvaccinated cattle and a case fatality rate of 24% (Abutarbush, 2014). The low apparent case fatality rate of 1.2% found in this outbreak could be a true low case fatality rate, or could be a result of poor follow-up of cases by field veterinarians or under-reporting to the SVCS.

Real-time PCR is a rapid, sensitive and specific method for confirmation of capripoxviruses including LSD (Balinsky et al., 2008). In this investigation, two-thirds of all samples tested from suspect animals were positive for the presence of viral DNA. Skin nodule samples consistently tested positive for LSDV; blood and organ samples were less likely to test positive. This aligns with the results of a study that found that LSD viremia is relatively short-lived – blood samples were positive for PCR for 4–11 days post-infection, while virus could be detected in skin lesions up to 92 days post-infection (Tuppurainen et al., 2005). In addition, on average, skin nodule samples in this study exhibited a higher concentration of virus than other samples, as evidenced by the lower average *C_T_* values observed in PCR testing. Quantitative real-time PCR assays were not performed, so the viral load of the different sample types could not be estimated.

Since the virus is very stable in the environment and can be transmitted by insects, mass vaccination of livestock is required to control the spread of disease (Wainwright et al., 2013; Tuppurainen and Oura, 2014). Other countries, such as Israel and Lebanon, have successfully controlled outbreaks with vaccination (Tuppurainen and Oura, 2014). Sheep and goat pox virus vaccines have been widely used against LSDV in cattle because the capripox viruses tend to be host-specific, yet offer cross-protection within the *Capripoxvirus* genus when vaccinations are administered (OIE Terrestrial Manual, 2010; Tuppurainen et al., 2014). The vaccines purchased for use in Azerbaijan’s vaccination program were advertised as a sheep–goat virus, which should offer cattle immunity against LSDV. Ongoing vaccination of cattle in the affected and surrounding areas will be necessary to keep cattle protected against exposure to the virus through the environment and biting insects. Vector control is also an important aspect of limiting spread of disease and should be used during active outbreaks.

Lumpy skin disease virus was detected for the first time in Bilasuvar rayon in Azerbaijan in 2014 and subsequently identified in three other rayons. Control measures were implemented, including restricted animal movement, vector control and a vaccination campaign. No additional cases were reported after November 2014. However, environmental persistence of the virus will likely continue to pose a risk to unvaccinated cattle in the affected rayons. Ongoing passive surveillance will continue to look for new cases throughout the country.

## Author Contributions

All authors listed, have made substantial, direct and intellectual contribution to the work, and approved it for publication.

## Conflict of Interest Statement

The authors declare that the research was conducted in the absence of any commercial or financial relationships that could be construed as a potential conflict of interest.
